# A case study of polypharmacy management in nine European countries: Implications for change management and implementation

**DOI:** 10.1371/journal.pone.0195232

**Published:** 2018-04-18

**Authors:** Jennifer McIntosh, Albert Alonso, Katie MacLure, Derek Stewart, Thomas Kempen, Alpana Mair, Margarida Castel-Branco, Carles Codina, Fernando Fernandez-Llimos, Glenda Fleming, Dimitra Gennimata, Ulrika Gillespie, Cathy Harrison, Maddalena Illario, Ulrike Junius-Walker, Christos F. Kampolis, Przemyslaw Kardas, Pawel Lewek, João Malva, Enrica Menditto, Claire Scullin, Birgitt Wiese, on behalf of the SIMPATHY Consortium

**Affiliations:** 1 Departament de Recerca i Innovació, Fundació Clínic per a la Recerca Biomèdica, Barcelona, Spain; 2 School of Pharmacy and Life Sciences, Robert Gordon University, Aberdeen, Scotland; 3 Pharmacy Department, Uppsala University Hospital, Uppsala, Sweden; 4 Effective prescribing and therapeutics, Health and social care directorate, Scottish Government, Edinburgh, Scotland; 5 Laboratory of Pharmacology and Pharmaceutical Care, Faculty of Pharmacy, University of Coimbra, Coimbra, Portugal; 6 Servei de Farmàcia, Hospital Clínic de Barcelona, Barcelona, Spain; 7 Institute for Medicines Research, Department of Social Pharmacy, Faculty of Pharmacy, University of Lisbon, Lisboa, Portugal; 8 Pharmacy Department and Regional Medicines Optimisation Innovation Centre(MOIC) Northern Health and Social Care Trust, Antrim, Northern Ireland; 9 Department of Social and Education Policy, University of Peloponnese, Korinthos, Greece; 10 eHealth Innovation Unit, 1^st^ Regional Health Authority of Attica, Athens, Greece; 11 Department of Health, Belfast, Northern Ireland; 12 Federico II University Hospital, Naples, Italy; 13 Institute of General Practice, Hannover Medical School, Hannover, Germany; 14 Department of Family Medicine, Medical University of Lodz, Lodz, Poland; 15 Institute of Biomedical Imaging and Life Sciences (IBILI) and Institute of Pharmacology and Experimental Therapeutics, Faculty of Medicine, University of Coimbra, Coimbra, Portugal; 16 CIRFF, Center of Pharmacoeconomics, University of Naples Federico II, Naples, Italy; 17 Clinical & Practice Research Group, School of Pharmacy, Queen’s University, Belfast, Northern Ireland; University of Basel, SWAZILAND

## Abstract

**Background:**

Multimorbidity and its associated polypharmacy contribute to an increase in adverse drug events, hospitalizations, and healthcare spending. This study aimed to address: *what* exists regarding polypharmacy management in the European Union (EU); w*hy* programs were, or were not, developed; and, *how* identified initiatives were developed, implemented, and sustained.

**Methods:**

Change management principles (Kotter) and normalization process theory (NPT) informed data collection and analysis. Nine case studies were conducted in eight EU countries: Germany (Lower Saxony), Greece, Italy (Campania), Poland, Portugal, Spain (Catalonia), Sweden (Uppsala), and the United Kingdom (Northern Ireland and Scotland). The workflow included a review of country/region specific polypharmacy policies, key informant interviews with stakeholders involved in policy development and implementation and, focus groups of clinicians and managers. Data were analyzed using thematic analysis of individual cases and framework analysis across cases.

**Results:**

Polypharmacy initiatives were identified in five regions (Catalonia, Lower Saxony, Northern Ireland, Scotland, and Uppsala) and included all care settings. There was agreement, even in cases without initiatives, that polypharmacy is a significant issue to address. Common themes regarding the development and implementation of polypharmacy management initiatives were: locally adapted solutions, organizational culture supporting innovation and teamwork, adequate workforce training, multidisciplinary teams, changes in workflow, redefinition of roles and responsibilities of professionals, policies and legislation supporting the initiative, and data management and information and communication systems to assist development and implementation. Depending on the setting, these were considered either facilitators or barriers to implementation.

**Conclusion:**

Within the studied EU countries, polypharmacy management was not widely addressed. These results highlight the importance of change management and theory-based implementation strategies, and provide examples of polypharmacy management initiatives that can assist managers and policymakers in developing new programs or scaling up existing ones, particularly in places currently lacking such initiatives.

## Introduction

Morbidity patterns are shifting towards chronic disease [1, 2], and their management has become a major priority for health systems around the world. Particular concerns arise in patients with multimorbidity, defined as the coexistence of two or more chronic conditions in the same individual [3]. Providing care for these patients has a significant impact on health systems and societies; they utilize more health services [4, 5], are at increased risk of disability [6], report lower quality of life [7], and die prematurely [8]. Older adults, aged 65 and over, are more likely to experience multimorbidity [9], although it is increasingly seen in younger patients [10], and is growing in prevalence [11, 12].

One consequence of multimorbidity is polypharmacy, commonly defined as taking five or more medications [13]. Polypharmacy has been described as “one of the greatest prescribing challenges,” [14]. It should be noted that polypharmacy is not always negative, and in many cases, is the best option for a patient. Therefore, there is rationale for referring to appropriate polypharmacy (optimal prescribing of multiple medications) or inappropriate polypharmacy (prescribing multiple medications where the potential harms outweigh the benefits), with less emphasis on the number of medications a patient is taking [15]. However, polypharmacy does increase the likelihood of adverse drug events [16, 17], drug interactions, drug-related hospitalizations [18], contributes to non-adherence [19], and higher health care costs [20]. Polypharmacy has been increasing in recent years, due to the rise in the prevalence of multimorbidity and an emphasis on single disease clinical practice guidelines on one hand [21, 22], as well as prolonged life span, and better screening, on the other. For all these reasons, polypharmacy is considered a growing public health issue that needs to be addressed by health care policymakers throughout the world, including those of the European Union (EU) [23].

Although the impact of inappropriate polypharmacy on health and economic outcomes is clear, addressing it remains a challenge. There are gaps in the literature regarding care of older patients with polypharmacy, resulting from the exclusion of older adults from clinical trials, lack of focus in clinical practice guidelines on issues such as screening and prevention in older patients, and lack of agreed guidance on the treatment of advanced disease near the end of life [24]. A meta-analysis evaluating pharmacist interventions in older adults showed improvements in therapeutic outcomes, safety, hospitalizations and adherence, although there was significant variability between studies [25]. Similarly, systematic reviews by Patterson [26] and later updated by Cooper [27] comparing interventions to address polypharmacy in older patients reported that pharmaceutical care improves prescribing, although there is still uncertainty about what elements make an intervention successful. In addition, the question of how to successfully implement new polypharmacy management practice models across the full care continuum has yet to be answered [28].

Project SIMPATHY (Stimulating Innovation Management of Polypharmacy and Adherence in the Elderly) sought to address the issue of inappropriate polypharmacy and related non-adherence in older patients across the EU by stimulating and supporting innovation around polypharmacy management. Co-funded by the European Union’s Health Programme, project SIMPATHY began in June of 2015 and concluded in May of 2017 and was led by the Scottish Government. The SIMPATHY Consortium was composed of a range of stakeholders including physicians, pharmacists, health policy makers, health economists, and academic researchers.

To achieve its goal, a series of three interrelated work streams were conducted with outputs that included: case studies of the management of polypharmacy; a systematic review of EU polypharmacy policies and guidelines; an EU benchmarking survey; a Political, Economic, Sociocultural, Technological, Environmental and Legal (PESTEL) analysis [29]; plus an analysis of the Strengths, Weaknesses, Opportunities, and Threats (SWOT) in consortium countries; and, an EU-wide modified Delphi survey to validate findings [30, 31]. This report presents the findings of the case studies.

The aims of the case studies were to address: *what* exists regarding polypharmacy management initiatives in the EU; w*hy* they were or were not developed in the current form (the underlying rationale); and, *how* any identified initiatives were developed, implemented, and sustained.

## Methods

### Case study design

Case study methodology is a set of procedures that can be applied systematically to provide an in-depth understanding of a specific focus of interest [32]. This design was selected to facilitate the holistic examination of both the clinical practice and the context surrounding the development and implementation of polypharmacy management initiatives.

**Fig 1:**
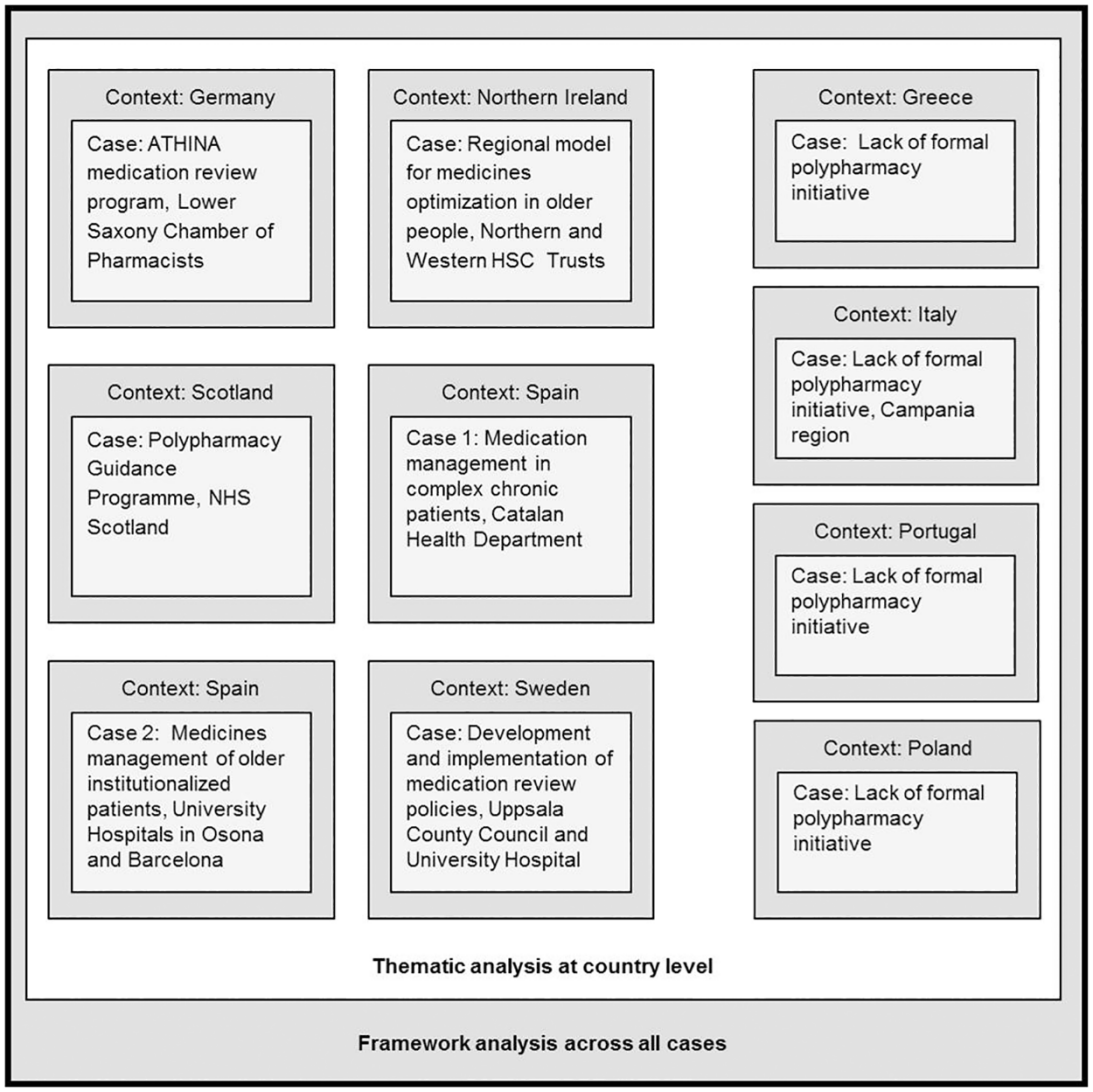
Case study design. HSC: Health and Social Care; NHS: National Health Service.

The eight EU SIMPATHY consortium countries (Germany (Lower Saxony), Greece, Italy (Campania), Poland, Portugal, Spain (Catalonia), Sweden (Uppsala), and the United Kingdom (Northern Ireland and Scotland)) were studied using a holistic multiple-case study design [31]. These European countries were purposefully selected to provide geographic diversity to the SIMPATHY consortium. In case study sites where researchers identified a polypharmacy management initiative, the initiative (which could be a local, regional, or national initiative) served as the case and the context for analysis was the country. In sites where no polypharmacy management initiative was identified, the case was an analysis of the reasons for not having an initiative, and the context was the country (Fig 1). Polypharmacy management initiatives were selected based on the knowledge and expertise of local research teams.

### Underlying theories and models

There are multiple theories, models, and frameworks in the literature regarding both the implementation of new clinical practices and leading organizational change [33, 34]. In order to gain insight into both the change management that drove, and actions that embedded, polypharmacy initiatives into daily practice, we selected one change management model (Kotter’s Eight Steps Process for Leading Change (Kotter)) and one theory of implementation (Normalization Process Theory (NPT)) to inform both the data collection and analysis [35, 36]. Kotter describes eight steps to managing change including: creating a sense of urgency; building a guiding coalition; forming a guiding vision; enlisting a volunteer army; enabling action by removing barriers; generating short term wins; sustaining accelerations; and instituting change. NPT has been used to evaluate the implementation of a broad range of complex health care practices [37–40], and consists of four domains: coherence, cognitive participation, collective action, and reflexive monitoring. Together, Kotter and NPT provide a rigorous and robust lens for exploring cases with, and without, polypharmacy management initiatives.

### Data collection

Data collection in each country occurred in three phases: a desk review of published and grey literature on polypharmacy management; key informant interviews with experts involved in the development and implementation of polypharmacy management initiatives; and focus groups to triangulate findings from both the desk reviews and key informant interviews. Within each country, a research team consisting of at least one member of the SIMPATHY consortium was responsible for both data collection and analysis at the local level.

All study participants were provided with an information sheet outlining the specific study aims and project SIMPATHY in general prior to participating in either the interviews or focus groups. Written consent for participation and audio-recording was obtained. All interviews and focus groups were audio-recorded, transcribed verbatim and analyzed in the local language, although the final reports were prepared in English. All data was stored on password protected computers and participant identities were kept anonymous in all reports. All local institutional review boards were consulted for any ethical issues. Specific approval was provided by the following ethics boards: Comité Ético de Investigación Clínica del Hospital Clínic, Barcelona, Spain; MHH Ethik-Kommission, Hannover, Germany, and; Comissão de Ética da Faculdade de Medicina da Universidade de Coimbra, Coimbra, Portugal. In all other study sites, there was no need for formal ethics approval as it was either seen as part of service development and quality improvement, or it did not require official review due to the lack of involvement of patients and there was no experimental design.

#### Desk review

Using a structured guide specifically developed for this study, the desk review was conducted by local research teams between September 2015 and October 2015. The guide was designed to identify published documents that would shed light on the development, implementation, and sustainability of any country/region specific polypharmacy management initiatives. It was developed by the case study coordinators (JM and AA) and reviewed by members of the SIMPATHY consortium as well as by experts outside the consortium for completeness. Each local research team answered a series of questions within the guide on the following topics related to the development and implementation of polypharmacy management initiatives: impact of external economic pressures on the health system; the role of the government; roles of non-governmental organizations; overview of relevant points of the healthcare system (e. g. financing, decision making, responsibilities of healthcare professionals); health information and communication capacity (population level monitoring, electronic medical records, electronic prescribing); and, details of implementation of a polypharmacy initiative at the institutional level. Documents used to provide data for the desk review included peer-reviewed publications, government or institutional policies, clinical practice guidelines, laws and regulations, and government reports.

#### Key informant interviews

After the desk review was completed, key informants with knowledge of the development, implementation, and ongoing evaluation of the initiative were recruited by the local research team. In cases where no polypharmacy initiatives existed, key informants were those familiar with existing medication management policies and able to speak to any current and future policy initiatives. Targets for recruitment included at least one key informant from each of the following groups: policymakers overseeing development of polypharmacy management policies; health system managers responsible for implementation at the institution level; and healthcare providers (including physicians, pharmacists, nurses, or other patient educators). The exact profile mix was determined by researchers within each case study site and informed by the results of the desk review. To ensure appropriate representation of stakeholder groups, a proposed list of interviewees and rationale for selection was reviewed by the study coordinators.

Interviews were conducted either face-to-face or by telephone between November 2015 and January 2016 in the local language using a semi-structured interview guide and lasted approximately 60 minutes. The interview guide was based on principles from Kotter and NPT, previously listed, and addressed the rationale for development of the initiative; implementation strategies; integration into the daily work flow; evaluation; and, plans for future developments. The guide was reviewed by members of the SIMPATHY consortium both via email and through phone conversations prior to data collection. Interviewers received both in-person and web-based training on conducting interviews and in using the guide.

#### Focus groups

Findings from both the desk review and interviews were analyzed and the major themes from each site were drawn from these data. To confirm the trustworthiness of these conclusions, identify any gaps or weaknesses in the reports, and fine tune the final messages, each research team conducted one focus group between February 2016 and March of 2016 to review the report findings. Focus group recruitment followed the same process as the key informants. Key informants who had previously been interviewed were eligible for inclusion in the focus group, and as with the interviews, the final mix of profiles depended on the findings from individual case studies. The target number of participants for the focus groups was between five and seven people and was scheduled to last for 60–90 minutes. The focus groups were run by members of the research team with one moderator and one note taker. Each moderator used a topic guide developed by the study coordinators that was reviewed using the same process as the interview guide. The topic guide included questions about how the results in the report matched with personal experience and with what they know of polypharmacy management in their area, if there were any points that had been missed or not emphasized enough, and if there was anything incorrect in the report. As with the interviews, moderators also received both in-person and web-based training. See S1 File for additional information on data collection tools.

### Data analysis

Research teams within each country produced a summary report including a narrative summary of their desk review, analysis of the interview and focus group data, as well as a time series analysis of key events, and a summary of lessons learned. This was based on the questions in the desk review topic guide, and a thematic analysis [41] of the interviews using a deductive coding framework based on Kotter and NPT, pre-agreed by the consortium partners. In addition to the deductive coding, there was an element of inductive coding, as new themes arose that did not fit within the predetermined framework. Consistency in applying the codes was promoted through consortium-wide training via an in-person workshop and follow up conference calls to discuss questions that arose during the analysis process. Individual case study reports were written in English using a standardized template to promote consistency in the type, style and reporting of findings. The study coordinators used these reports to conduct a framework analysis [42] to generate the summary findings. Each study coordinator independently reviewed the country level reports and placed major themes within the predefined framework, which was again based on Kotter and NPT.

## Results

### Characteristics of polypharmacy initiatives

Five sites had polypharmacy initiatives sufficiently developed for inclusion: Spain (Catalonia), Germany (Lower Saxony), Sweden (Uppsala), and both of the United Kingdom sites (Northern Ireland and Scotland). Two distinct programs were identified for inclusion in Catalonia. Four sites, Greece, Italy (Campania), Poland, and Portugal, did not identify any initiatives or policies related to polypharmacy, although at the time the study was completed Poland was in the process of developing a medication use policy that will likely include polypharmacy management based on a pharmaceutical care model. Tables 1 and 2 provide a summary of the range of program scope, targets, and objectives in countries with polypharmacy management initiatives.

**Table 1:** Summary of identified polypharmacy management initiatives.

Country (region)	Scope; Setting	Patients Targeted for Intervention	Healthcare Provider	Program Objectives	Description of Activities
Spain (Catalonia)^1^	Regional; Primary care	Those meeting health system definition of complex chronic disease	Primary care physicians	Improve 1) Patient safety and reduce drug related problems; 2) health outcomes and control of chronic disease; 3) Adherence and; 4) Healthcare quality and patient quality of life	Complex chronic patients flagged in electronic medical record Physicians required to review all flagged patients according to guidance published by the Catalan Health Department
Spain (Catalonia)^1^	Regional; Institutional	Admitted to acute geriatric unit	Geriatrician and hospital pharmacist	Improve global patient health and well-being	Therapy goals established with patients and families Pharmaceutical care plan developed accounting for specific diagnosis, the indication for each medication (therapeutic, primary or secondary prevention), and the life expectancy of the patient Care plans shared via fax or verbally with primary care physicians
Germany (Lower Saxony)	Regional pilot; Community pharmacy	Determined by pharmacists but usually ≥ 5 medications and ≥ 60 years old	Community pharmacist	1) minimize drug risks; 2) improve efficacy of pharmaceutical care; 3) improve adherence and; 4) communicate findings with physicians	Pharmacists choosing to participate undergo training through their professional organization sponsoring the activity During two separate patient visits pharmacists conduct a comprehensive medication review including adherence counselling Results are communicated to patients who may choose to share these with their physician or with patient consent directly to doctors

^1)^Two different programs were identified in Catalonia, a government sponsored primary care model and an institutionally sponsored model including inpatient hospital care, long-term care and nursing homes;

^2)^ includes step-down facilities providing time-limited services geared towards reducing prolonged hospital stays and promoting independence;

^3)^ Scotland has estimated the risk of emergency hospital admission for its population;

^4)^ Sweden has national legislation mandating medication reviews take place but the setting is determined by local health authorities depending on their resources and need

**Table 2:** Summary of identified polypharmacy management initiatives (continued).

Country (region)	Scope; Setting	Patients Targeted for Intervention	Healthcare Provider	Program Objectives	Description of Activities
United Kingdom (Northern Ireland)	Regional; Intermediate care^2^	Admitted to intermediate care	Case management and consultant pharmacists	Develop, test and scale up a regional model for Medicines Optimization in older people	Pharmacists working in intermediate care setting are supported by a senior consultant pharmacist in developing patient-centered pharmaceutical care plans Medication adherence assessed Care plans communicated with general practitioner Patient followed by pharmacist post-discharge for up to 30 days
United Kingdom (Scotland)	National; Care homes, Primary care	All patients in care homes aged 50 or older patients 75 and older, on 10 or more medications (one must be high risk) and at high risk for hospital admission	Pharmacists and physicians in primary care	Systematically address inappropriate polypharmacy and adherence across Scotland in order to minimize harm, optimize benefits, reduce hospitalizations and medication waste^3^	Apply medication review according to processes outlined in National Polypharmacy Guidance Guidance addresses establishing shared treatment goals, evaluating value of medications based on number needed to treat, balances disease state recommendations with life expectancy, and adherence
Sweden	National; Multiple^4^	Patients aged ≥ 75 with 5 or more prescription drugs	Physicians or teams of physicians and clinical pharmacists	Increase and ensure the quality, safety and sustainability of pharmaceutical care, focusing on polypharmacy in the elderly	Guidance and tutorial on performing medication reconciliation and comprehensive medication reviews published by the National Board of Health and Welfare, although application is optional Goal of guidance is to optimize the patient’s medication treatment and to minimize the incidence of drug-related problems. Changes made based on guidance should be are communicated orally and in writing to patients and other healthcare providers

^1)^ Two different programs were identified in Catalonia, a government sponsored primary care model and an institutionally sponsored model including inpatient hospital care, long-term care and nursing homes;

^2)^ includes step-down facilities providing time-limited services geared towards reducing prolonged hospital stays and promoting independence;

^3)^ Scotland has estimated the risk of emergency hospital admission for its population;

^4)^ Sweden has national legislation mandating medication reviews take place but the setting is determined by local health authorities depending on their resources and need

### Participant characteristics

**Fig 2:**
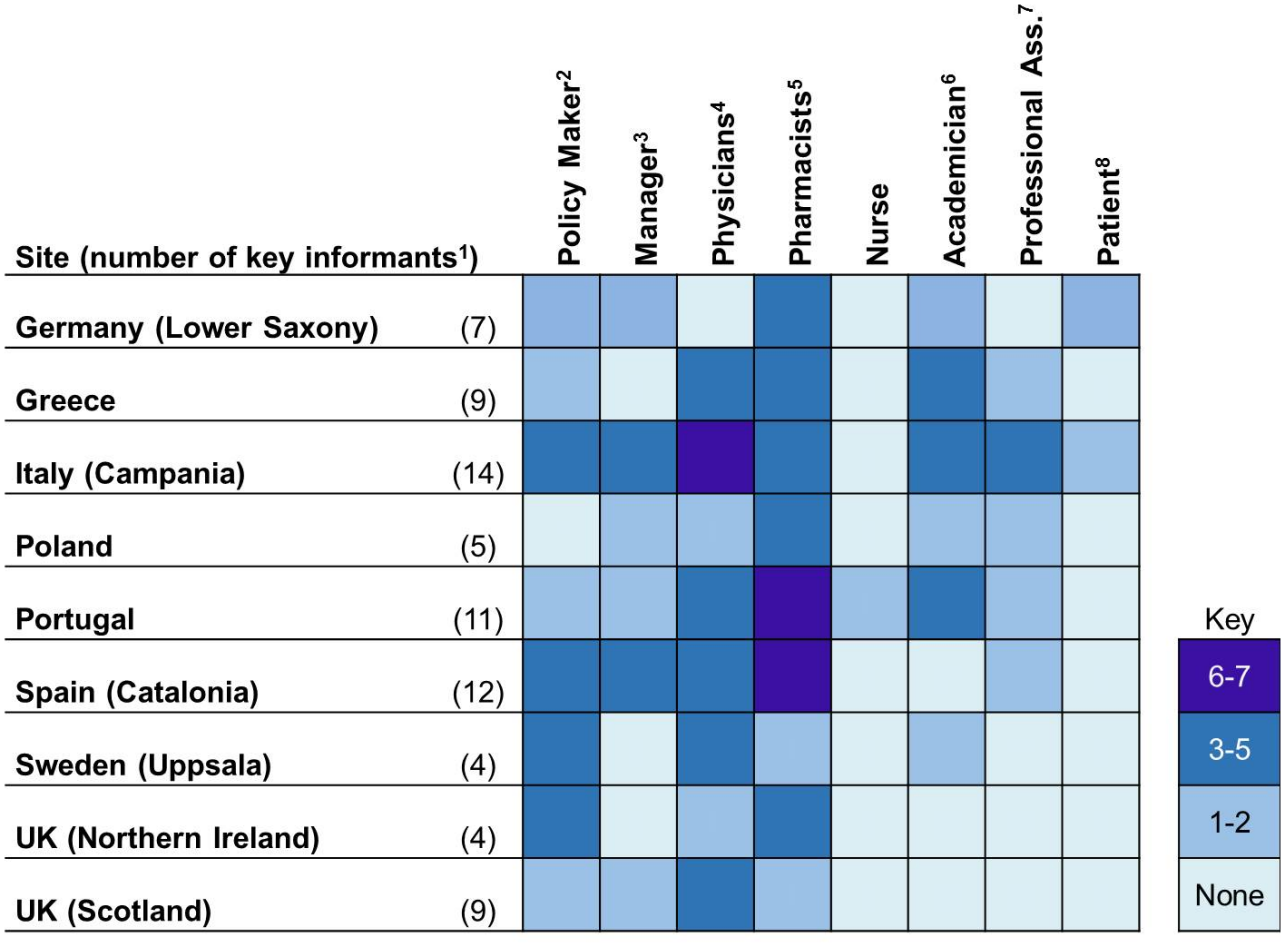
Key informant interview characteristics. 1) The total number of key informants and the total number of profile characteristics are not equal, as more than one characteristic could be applied to the same key informant (e.g. one informant could be both a physician and a manager); 2) Those working in governing bodies or agencies overseeing health systems at a regional or national level; 3) Includes hospital CEO’s, primary care center directors, and department managers; 4) Geriatricians, hospitalists, general practitioners; 5) Hospital, primary care, and community; 6) Departments of pharmacy and medicine, also includes research and clinical faculty; 7) Medicine and pharmacy; 8) Patients and representatives of patient associations. UK: United Kingdom.

**Fig 3:**
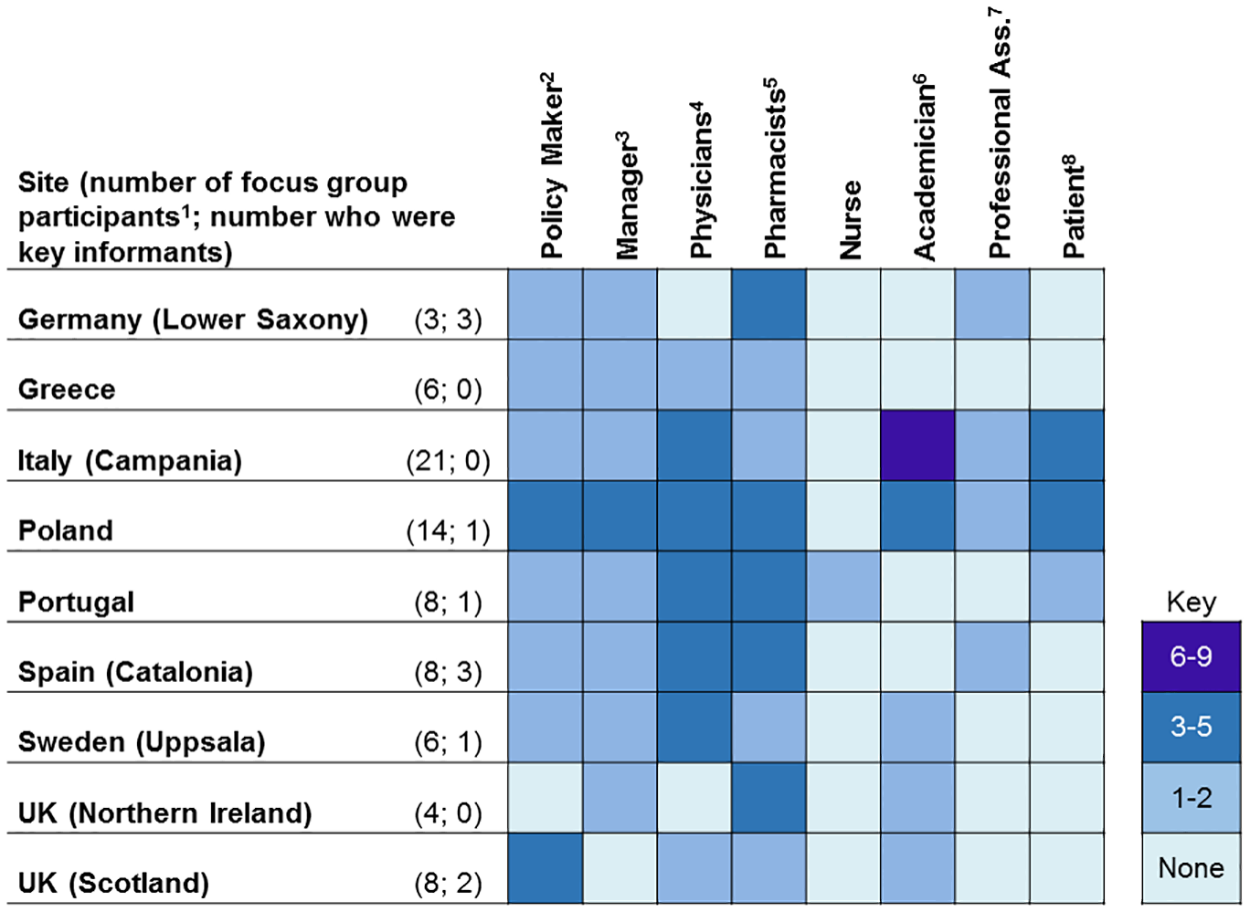
Focus group participant characteristics. 1) The total number of key informants and the total number of profile characteristics are not equal, as more than one characteristic could be applied to the same key informant (e.g. one informant could be both a physician and a manager); 2) Those working in governing bodies or agencies overseeing health systems at a regional or national level; 3) Includes hospital CEO’s, primary care center directors, and department managers; 4) Geriatricians, hospitalists, general practitioners; 5) Hospital, primary care, and community; 6) Departments of pharmacy and medicine, also includes research and clinical faculty; 7) Medicine and pharmacy; 8) Patients and representatives of patient associations. UK: United Kingdom.

Across the nine case study sites a total of 75 interviews and 12 focus groups, with a total of 74 participants, were conducted. (In Poland three and in Italy two simultaneous focus groups were conducted to facilitate conversation between policy makers and managers, healthcare providers, and patients.) Figs 2 and 3 summarize key informant interview and focus group participant characteristics by country and category. Categories are intentionally broad to protect the identity of key informants.

### Characteristics of future polypharmacy initiatives

Amongst the case studies with no polypharmacy management and adherence initiatives, key informants identified criteria for future initiatives which were similar to those of existing programs. Most agreed any initiatives should target older patients with frailty, as well as patients of any age with multiple chronic conditions. There was also agreement that a multidisciplinary approach should be employed. Physicians and pharmacists were the most commonly mentioned health professionals to implement medication reviews, a fundamental part of polypharmacy management. Similar to countries with existing initiatives, multiple settings for implementation were mentioned including community pharmacies, primary care, and hospitals. In Poland and Greece community pharmacies were seen as a particularly important setting in which to address polypharmacy.

### Common themes across case study sites

The framework analysis across case studies revealed a number of themes that were common to both cases with and without polypharmacy initiatives. Table 3 lists the themes as they relate to NPT and Kotter, and are detailed below. Depending on the setting, each of these themes could either be a facilitator or barrier to implementation of a polypharmacy initiative.

**Table 3:** Major themes categorized by NPT and Kotter.

Theme	NPT Construct	Kotter’s Steps
Global pressures with local solutions	Inducted unmatched theme	Inducted unmatched theme
Aligning polypharmacy with other health policy initiatives	Coherence	Build a guiding coalition Form strategic vision and initiatives
Organizational culture can help or hinder	Coherence supporting collective action	Enable action by removing barriers
Need for strong data management and ICT	Collective action Reflexive monitoring	Creating urgency Generate short term wins Sustain acceleration
The role of training the workforce	Coherence Cognitive participation Collective action	Enable action
Leading change by sharing leadership	Cognitive participation	Build a guiding coalition
Creating and implementing multidisciplinary teams	Cognitive participation Collective action	Build a guiding coalition; Form strategic vision and initiatives
Strategic alignment of financial and human resources	Collective action	Remove barriers; Institute change
Strategic policies and legislation	Coherence	Form strategic vision and initiatives; Institute change

ICT: information and communication technology

#### Global pressures with local solutions: “We have to start asking ourselves how we are going to treat [the geriatric population] which will be much larger in 30 years.” Spanish hospital pharmacist

All case study participants described global, macro level issues that served to support or hinder the development of polypharmacy initiatives. Amongst these, two common pressures identified were the need to improve efficiency in the health system and the need to prepare for an ageing population and subsequent increased complexity of care. In some situations, this contributed to a sense of urgency, spurring the development of polypharmacy initiatives. However, in some case study sites without initiatives, Italy (Campania), Portugal and Greece, pressure to improve economic efficiency was too extreme, and instead of driving change, created an obstacle. As expressed by a Greek pharmacist, “the main goals for the hospital are financial” and polypharmacy management was seen as an added “luxury.”

Although each case described the same pressures at the macro level, solutions varied based on the available resources and culture within the healthcare system. Illustrating the need for local solutions to the universally identified challenges, a Portuguese physician said, “We cannot make an accreditation like Northerners based on checklists. That will never work with us, we are not Scottish.”

#### Aligning polypharmacy with other strategic initiatives: “If polypharmacy just sat as a stand-alone prescription project, it would have got lost.” Scottish pharmacist manager

Of the polypharmacy management initiatives identified, all were developed within the context of larger initiatives, such as an increased focus on older, more complex patients, medication safety, or workforce development. In discussions regarding workforce development, expanding the role of pharmacists was specifically mentioned in multiple cases. In Germany, the Chamber of Pharmacists (the professional association) saw the development of a polypharmacy management initiative as an opportunity to both improve the safe use of medications (a national priority) and for pharmacists to “positively position themselves as a health professional” (a priority of the Chamber). Aligning polypharmacy management with other strategic initiatives contributed to coherence, or shared understanding, and facilitated the creation of guiding coalitions and a shared vision.

#### Organizational culture can help or hinder: “There is no sense of teamwork in healthcare, medical doctors are not accustomed to cooperation and collaboration.” Greek policymaker

Organizational culture was identified by sites with and without polypharmacy management initiatives as either supporting cognitive participation and subsequent collective action, or as a barrier that impedes change. In sites with polypharmacy management initiatives, there was often a culture of innovation, such as in Northern Ireland, where the Department of Health and Medicines Optimisation and Innovation Centre described itself as “outward looking,” or in Catalonia where a hospital CEO described his job as “managing innovation.” In contrast, in multiple sites without initiatives, the absence of a culture of teamwork was mentioned repeatedly, such as in Poland, where a pharmacist stated, “Physicians are unwilling to solve the issue when it is transferred [from a pharmacist to a physician] by a patient.” Changing organizational culture and improving interprofessional communication were seen as prerequisites for implementation in Portugal.

#### The need for strong data management and information and communication technology (ICT): “One of the most important things is to start measuring drug use, to get a view of the situation and make healthcare professionals aware of the problem of inappropriate polypharmacy.” Swedish geriatrician

Consistent across case studies was the need for data management systems with the capacity to quantify the extent and consequences of polypharmacy at a population level, and integrated and networked ICT systems to facilitate implementation and monitoring at the clinical level. Data management systems were identified in cases with and without initiatives as critical to generating a sense of urgency and contributing to coherence. ICT systems also contributed to collective action by facilitating implementation. This was the case in Catalonia where a primary care manager stated, “One advantage of our system is that it is completely computerized, you can register everything, and one thing we have registered is if the patient’s medications have been revised.” Countries or regions without ICT systems robust enough to monitor polypharmacy, Lower Saxony (Germany), Poland, and Portugal, identified this as a “main barrier” to implementation stating that there is an “urgent need” for ICT systems across sectors that support polypharmacy management.

#### The importance of training the workforce: “Medical education [at the university level] is not structured and adequate to address the issue of polypharmacy,” Italian physician

Education was also identified as both a facilitator and barrier to implementation. Where present, education and training contributed to coherence by creating a common understanding of the problem. In Scotland and Sweden this was achieved by incorporating polypharmacy management into the undergraduate training of both pharmacists and physicians. Conversely, key informants in Italy, Portugal, and Germany all noted that inadequate training was a barrier.

In addition to coherence, education and training also contributed to cognitive participation by helping each team member identify their role, and facilitated collective action by ensuring those responsible for implementation had the requisite skills. Having an agency tasked with continuing professional development, as was the case in Northern Ireland and Scotland (UK), was noted as instrumental in developing the workforce required for their initiatives.

#### Leading change by sharing leadership: “You need knowledge of change management to do this.” Swedish physician and policymaker

Multiple key informants in case studies with and without polypharmacy management initiatives identified the need for strategic planning and for leadership at both the management and clinician levels. The importance of leadership at the clinical level was stressed by a Scottish senior manager who said, “it [the polypharmacy management program] wasn’t imposed on anybody—the clinicians led it.”

#### Creating and implementing multidisciplinary teams: “Teamwork is not easy: we need to find a trade-off between hierarchy and teamwork.” Italian physician

The role and importance of multidisciplinary teams was echoed across the case studies, and the design and implementation of these teams mainly fell under cognitive participation and collective action. Creating a team often involved redefining roles of different healthcare providers (a component of cognitive participation), a task that proved challenging and sometimes resulted in tension between different professional groups. A Swedish physician addressed this tension between pharmacists and physicians noting that, “there are also still some voices against the performance of [medication] reviews by a pharmacist.” Pharmacists in Germany were hopeful that their program would result in “more cooperation on the doctor-pharmacist level” but were ultimately concerned that their new role might be viewed as trying to “usurp” the role of physicians. In contrast, in Catalonia where the goal of the hospital based program was to “design the patient’s prescription in a shared way between physicians and pharmacists” there was little or no tension. In Scotland, outcomes data relieved initial concerns of physicians that pharmacists were not “able to do the [medication] reviews as well as a general practitioner,” and in Italy key informants suggested that training and shared clinical objectives could be strategies to promote team building.

#### Strategic alignment of financial and human resources: “We will need to change the dynamic of how we work if we want to do this.” Spanish primary care pharmacist

Reallocating resources to align with polypharmacy management objectives was clearly expressed in all cases, and can be seen both as an aspect of collective action and a way to facilitate change by removing barriers. One strategy described in multiple case studies to redistribute human resources was explicitly devoting physician and pharmacist time to polypharmacy management. When there was no change in workflow and inadequate time dedicated to a new initiative, such as in the community pharmacy initiative in Lower Saxony (Germany), implementation was a challenge. Pharmacists there described doing medication reviews “in their spare time” with one pharmacist stating, “I have not integrated it correctly.”

The need to align payment systems, contracts, and pay-for-performance incentives with polypharmacy initiatives was noted in all cases, and a mix of these strategies were seen in the majority of cases with polypharmacy initiatives. In Germany, community pharmacists did not get reimbursed by insurance for their services, and this was seen as a barrier to implementation, even though providing the service resulted in professional satisfaction. In countries with no initiatives, key informants echoed the sentiments of a Greek policymaker who said that “more personnel should be hired, or the existing personnel should be better reimbursed for offering additional services.” Similarly, an Italian pharmacist noted that, “an adjunctive payment system could be a facilitator of a dedicated [polypharmacy management] service.”

#### Strategic policies and legislation: “[Polypharmacy management] is linked in with Transforming Your Care, the main policy direction in Northern Ireland.” Northern Irish lead pharmacist

Legislation regulating practice, such as the inclusion of medication reviews under pharmacists’ scope of practice in Germany or granting prescribing rights to non-physicians in the UK, provided a legal foundation for polypharmacy management in these cases. In Sweden, legislation was passed specifically requiring medication reviews for patients aged 75 or older taking five or more medications, thereby codifying the management of polypharmacy. Multiple case studies without initiatives expressed the need for central guidance and policies directing polypharmacy management. In Poland, the importance of EU policies was mentioned by a member of the Chamber of Pharmacy noting the need to “harmonize” policies throughout Europe.

Policies setting strategic goals for health systems were also credited with supporting the development of polypharmacy initiatives in all case study locations. These included pharmacy specific policies, such as Prescription for Excellence in Scotland, as well as general medications policies, such as the Action Plan for Medication Safety in Germany and the Medicines Optimization Quality Framework in Northern Ireland, or general health systems policies, like the Catalan Health Plan. In Poland accreditation standards for hospitals state that the “frequency of polypharmacy cases” must be monitored, which suggests polypharmacy management is performed in accredited hospitals on a regular basis, although no descriptions or evaluations of these activities were published to date.

## Discussion

As part of project SIMPATHY, case studies were conducted in an attempt to describe: what exists regarding polypharmacy management initiatives; why these programs were developed in the form they exist; and, how these programs were or were not developed, implemented, and sustained. A broad range of polypharmacy initiatives across multiple healthcare settings were identified and, in almost half of case studies, no formal polypharmacy management activities were noted. Settings for identified polypharmacy management initiatives included community pharmacies, primary, intermediate, and acute hospital care settings. The role of different professionals also varied widely, with Scotland and to a certain extent Northern Ireland utilizing independent pharmacist prescribers, while implementation in Catalonia (Spain) and Uppsala (Sweden) focused on physicians and on integrating pharmacists into multidisciplinary teams. In contrast, in Germany pharmacists were introduced to the role of medication checks with little connection to the prescribing doctors.

Even with the variation in approaches to managing polypharmacy, common themes were identified around the development, implementation, and sustainability of initiatives. These were common across study sites with and without polypharmacy initiatives, and provide insight into how and why different programs were developed. The themes included the need to align polypharmacy with other health system initiatives, create multidisciplinary teams, ensure education and training is adequate, develop data management and ICT systems to support monitoring and implementation, reallocate health provider time, and ensure that policies, legislation, and payment mechanisms are in line with the goals and objectives of the initiative. These findings are unique within the literature addressing polypharmacy, as we not only described the variety of clinical practices to address polypharmacy, but also the strategies to design and implement sustainable solutions.

The range of practice settings identified in this case study are reflective of the variety of polypharmacy management initiatives described in the literature [24, 25]. A meta-analysis evaluating pharmacist interventions in older adults in the United States included interventions in community pharmacy, ambulatory care, nursing homes and rehabilitation centers, and inpatient settings [25]. Likewise, a recent Cochrane review included interventions from primary care, nursing homes, and inpatient units, but none from community pharmacy [26].

### Polypharmacy: A complex intervention requiring a systems approach

The breadth of common themes identified and the variety of practice settings point to the fact that polypharmacy management is a complex intervention [43]. Therefore, these findings should be considered within the context of other complex, system-level approaches to quality improvement in healthcare and not simply within the context of a single discipline or department. There is significant overlap between the SIMPATHY case study findings and recommendations from major quality improvement frameworks from around the world. The 2001 report from the Institutes of Medicine (IOM) on improving the quality of the US medical system, Crossing the Quality Chasm, recommends that organizations should redesign the care process, improve use of information technology, address knowledge and skill management, build effective teams, and incorporate performance and outcome measurements [44]. These echo the specific findings of the case studies around the need to rethink how work gets done, the role of information technology and data management, the need to address workforce training, the importance of multidisciplinary teams, and the role of contracts and pay for performance to drive change.

The SIMPATHY findings are also similar to the enablers of service delivery reform outlined by the Health Council of Canada which include 1) leadership at all levels, 2) policies and legislation articulating the vision, 3) capacity-building for health professionals, 4) promotion of organizational cultures that support innovation and risk taking, and 5) measurement and reporting to provide continuous feedback [45]. As with the IOM report, the specific findings of the case studies are reflected here, including the need for shared leadership, the role of policy and legislation in shaping initiatives and the role of organizational culture in promoting or stifling innovation.

Finally, when viewed through the lens of integrated care, a framework that is increasingly used to address multimorbidity, there is also considerable overlap. The Rainbow Model of Integrated Care (RMIC) outlines integration at the system, organization, professional, and clinical levels [46, 47], which are categories that were all broadly represented in the case studies. There was significant overlap with the realm of professional integration, with both the case studies and the RMIC taxonomy identifying the role of health professional training, a focus on collaboration and teams, and the importance of developing the professional roles of different providers. All of the commonalities identified between the findings from the SIMPATHY case studies and other widely used frameworks indicate that these findings should be both relevant and transferable to multiple settings.

Although there is considerable overlap between the SIMPATHY case studies and established quality improvement frameworks, there are also differences. Compared to the RMIC and its taxonomy, there is much more emphasis in the case studies on professional level integration, such as creating a shared vision, team building, and interpersonal characteristics. There was also less emphasis in the case studies on issues related to organizational level integration when compared to the RMIC. This may be a reflection of the level of maturity of the polypharmacy management initiatives and policies examined in this study. Most of those identified were relatively new, and therefore may still be focusing on implementation and the role of specific professions (such as pharmacists), and not on the larger institutional factors. This also highlights that, although polypharmacy management can be seen as a component of integrated care or larger quality improvement initiatives, there are elements unique to its implementation that need to be explicitly considered as managers and policymakers look to address this issue.

### Role of theory and change management frameworks to drive implementation

The case study findings also provide insight into how to support the implementation of complex innovative practices in different settings. The analysis based on Kotter’s principles identified a density of activity in the early stages of Kotter’s process, specifically around creating a sense of urgency, a strategic vision, and a guiding coalition, but less in the later stages of instituting change. Many of the countries without initiatives identified clear barriers to achieving these crucial first steps (such as lack of data to create a sense of urgency), indicating that a change management framework might be useful in promoting the development of a polypharmacy initiative in these settings.

Analysis based on the NPT constructs provided valuable information regarding implementation that would not have surfaced within Kotter’s change management principles. The construct of collective action provided a significant wealth of information regarding the need for the reallocation of resources (both financial and personnel) as well as the challenges of creating and building multidisciplinary teams. These lessons will be particularly useful to those ready to move from policy to practice, or to those wishing to refine an existing practice. Together, both NPT and Kotter provided complementary ways of addressing polypharmacy management.

Although many of the lessons from the SIMPATHY case studies have been discussed in the context of quality improvement, these principles have not previously been systematically applied to published polypharmacy management interventions. To date, interventions and recommendations to address polypharmacy have been primarily focused at the clinical level looking, for example, at strategies to improve prescribing or patient adherence [48–50]. Many interventions either fail to take into account, or do not report on, broader systems level issues, such as the need to change reimbursement models or employ a change management strategy to speed implementation as was highlighted in this analysis. The similarities between the SIMPATHY case study findings and previously published systems level quality improvement recommendations indicate that, to most effectively address the issue of polypharmacy, a broader approach that goes beyond clinical level interventions is needed.

The findings from these case studies have practical implications for policymakers and managers that can be applied across the entire health system to address better polypharmacy management. These are applicable both to health systems with existing polypharmacy management initiatives, and those wishing to develop a new service. These include:

- Build on existing strengths. Those seeking solutions for polypharmacy management should aim to identify and utilize the strengths of the healthcare system within which they operate to benefit from existing professional roles, practices and service models.
- Create local solutions. The scope of programs identified highlights the importance of designing local solutions that take into account the existing infrastructure and resources, culture, and priorities of the health system. The range of initiatives and approaches identified within the case studies support the idea there is no blueprint or ‘one size fits all’ solution to addressing polypharmacy management, and that transferable elements within initiatives should be tailored to the meet local needs.
- Look for synergies. Polypharmacy management should be integrated into wider health policy improvement initiatives, nationally, regionally or locally to accelerate change and amplify the benefit to patients and the healthcare system.
- Foster a culture of innovation. Work should begin within the existing culture, but efforts should be made to promote a culture of innovation and quality improvement.
- Invest in data management and ICT. Data management and ICT systems that support monitoring, adherence, and implementation are crucial, and lack of these systems presents significant barriers to scaling up.
- Ensure education and training better prepares professionals to manage polypharmacy. Changes in undergraduate, postgraduate, and continuing professional development are needed to develop the multidisciplinary workforce necessary to address polypharmacy.
- Support development of leadership at all levels. Polypharmacy management is a system wide issue, and will require a change management strategy and shared leadership between policymakers, managers, and clinicians.
- Change how work gets done. Redesigning of workflow, including redistribution of work and responsibilities between healthcare professionals is essential to implementation.
- Employ multidisciplinary teams. The involvement of multidisciplinary teams employing a patient-centered approach facilitates the implementation of polypharmacy management.
- Assess current payment schemes. Reimbursement and payment incentives should be aligned with the goals and objectives of the initiative.
- Create the practice environment with legislation and policy. Legislation and policies are critical for defining the vision and creating the long-term regulatory environment to support polypharmacy management initiatives.

### Strength and limitations

The case study methodology provided a rich view of polypharmacy management initiatives in the EU. The combination of the desk review, key informant interviews, and focus groups provided a deeper understanding of the drivers and barriers to managing polypharmacy. The context for the cases included in this study were diverse in terms of geography, economies, sociocultural backgrounds, and covered a broad range of polypharmacy management initiatives, making the results transferable to multiple contexts.

The study did have some limitations. Researchers were given leeway in selecting key informants and focus group participants, and representation of different stakeholder groups was not uniform across the case study sites. This may have led to bias in data collection. Some healthcare providers who might play a role in polypharmacy management, such as nurses, were also underrepresented as key informants. This is partly due to pharmacists, due to their unique training, being seen as primary drivers in polypharmacy management. The framework analysis was based on the case study reports, and it is possible that there was inter-coder variation between sites, although we attempted to minimize this through in-person and online workshop training with researchers.

### Future directions

Given the clear agreement across all case study sites that polypharmacy is a pressing issue, and the alignment of the SIMPATHY findings with other quality improvement frameworks, future work should focus on theory based implementation. Researchers should look at the tools and methods from the field of implementation science and use frameworks such as the Consolidated Framework for Implementation Research [33] to guide future endeavors. Specific topics within the context of implementation include which reimbursement and incentive structures work best to facilitate implementation, what training methods are most successful in creating effective multidisciplinary teams, and how can organizational culture shift to support innovation.

## Conclusion

The policies and processes for addressing polypharmacy vary widely in the EU, and many countries in the EU are not formally addressing polypharmacy management. These case studies provide examples of initiatives that can be used by countries in the process of developing new polypharmacy management activities, as well as to those looking to scale up existing programs, and highlight the importance of change management and theory based implementation strategies [30].

## Supporting information

S1 File Data collection tools. (DOCX)
