# Supplementary material for: A case study of polypharmacy management in nine European countries: Implications for change management and implementation
Source: PLoS One. 2018 Apr 18;13(4):e0195232. doi: 10.1371/journal.pone.0195232 (PMC5905890; doi:10.1371/journal.pone.0195232)
Supplement: S1 File — (DOCX) [file pone.0195232.s001.docx]

# SIMPATHY Data collection tools

## Data sources

Data collection for case studies usually consists of a range of sources, commonly including archival information, interviews, and direct observation. SIMPATHY case study data collection will consist of three phases: a desk review, key informant interviews, and focus groups. Information on inclusion criteria and specific collection procedures are included in the individual sections. Below is a brief summary of each source. Of note, although direct observation would have provided significant insight into how a polypharmacy programme is truly operating, this would require resources beyond those available at the moment and therefore will not be used for this project.

##### Desk review

The primary focus of the desk review is to articulate the specific activities that take place as part of the polypharmacy programme. It will also provide an overview of the healthcare system and institutions within each case study, and describe some of the legislative or policy structures in place that support the polypharmacy programme. Existing public and internal documents will provide the data for this portion of the case study.

##### Key informant interviews

A second data source is the key informant interviews. Interviews will be used to add depth to the information collected in the desk review, while also providing insight into the development, implementation, and maintenance of the programme. The interviews will shed light on topics not specifically addressed in published documents, especially regarding management and leadership strategies to develop and sustain the programme. Some questions covered in the desk review may also be addressed in the interviews. Although we have attempted to minimize redundancies between the desk review and interviews, in some situations it is good, or even necessary, to utilize different data sources to look at the same issue in order gain a more accurate understanding the question at hand.

##### Focus groups

The third data source will be focus groups of patients, health care providers, and policy makers. The focus groups will be used to validate the findings generated be the desk review and key informant interviews. These discussions should let the research team know if their findings accurately reflect the experience of patients and practitioners in a real world setting.

# Desk Review

## Completing the desk review

### The research team

The initial phase of the case study is a desk review of policies and procedures outlining your polypharmacy programme. Almost all of these documents will be considered grey literature, and some may be internal working documents that are not available to the public. Therefore, it will be necessary for each case study team to identify a senior clinician (physician, pharmacist, nurse, etc.), policy maker, or both who is familiar with the development and implementation of the programme to assist with identifying relevant documents. This may be a member of the SIMPATHY research team, or may be someone from outside of the project, depending on the makeup of your current research team and the polypharmacy and adherence programme that you use for the case study. Identify one or two people who will assist in the desk review.

***Research Personnel Examples***

***Case Study from Spain***

**Senior Clinician:** Carles Codina is the head of the pharmacy departments in both the Hospital Clinic and the Vic University Hospital. He has a working knowledge of both the government and institutional policies and procedures that guide the polypharmacy programmes in Barcelona and Vic. Therefore, no additional personnel outside of the SIMPATHY team are required to complete the desk review.

**Research Staff:** Jennifer McIntosh is a contracted pharmacist who will complete the desk review utilizing publicly available documents and those provided by Carles Codina.

***Example case study from Scotland***

**Senior Clinicians:** Alpana Mair Deputy Chief Pharmacist for Scotland and Simon Hurding clinical lead for therapeutics for Scotland will undertake the desk review. They are responsible for advising on the policy at national level and also work with colleagues at NHS boards and have knowledge in order to gather the research data.

**Research Staff:** Moira Kinnear is a senior researcher in an NHS board who will complete the desk review accessing information that is available at health board level.

### Documents to include

The desk review should be completed utilizing existing published or internal documents. Include all policies that currently govern activities within your programme. Depending on the structure of your health care system, this may include national, regional, or local policies. In addition to publicly available documents, you may also include presentations, institutional policies, or published literature that describes the programme. All documents should be referenced appropriately with links to the original if available.

### Time frame

There is no limitation on the publication date of documents. That being said, all documents included should apply to the programme in its present form, so if more than one version of a policy is available, use the most recent version for the majority of the desk review. The only time an older version might also be used is to illustrate the time frame for developing the polypharmacy programme. In this case, older versions of a policy should be noted, but the content of the most recent version should be used.

Please note that you might not find every piece of information in the guide below in a written document. That unto itself is potentially interesting information, especially if key messages such as the rationale for developing a programme are not clearly outlined in a published guideline on the topic. Therefore, please note when you are unable to identify items below (instructions provided in the report template).Key informant interviews can also be used to identify or clarify topics in the desk review that are not addressed in published literature.

## Desk Review Guide

### Global issues

*The following questions refer to the general economic environment that surrounded the development of the polypharmacy programme.*

| ★ | *For countries without a mature or established polypharmacy programme, this section provides an opportunity to describe competing programmes influenced by their economic situation.* |
| --- | --- |

- How did wider economic issues affect health policy in your country or region? For example, did economics play a role in setting priorities for programmes to develop? Was cost containment or use of expensive medicines prioritised over other initiatives?
- Did these wider economic issues have any effect on the management of polypharmacy or the development of polypharmacy management programmes?

#### Healthcare system overview

*In this section please provide a description of the healthcare system in your country or region. This information can be addressed regardless of the presence or absence of a polypharmacy or adherence programme. Please specifically address the following points:*

- Financing: Is the financing public, private, or mixed? What type of out-of-pocket expenses are patients expected to cover?
- Decision-making: Where are decisions made regarding healthcare spending and policy? Is decision making devolved to local regions or is it centralized? If there are multiple levels of policy (national, regional, local) how are these integrated?
- Prescription medicines: How are prescription medicines financed? What is the role of community pharmacists in supplying medicines and how are they paid for this? Do patients ever have access to prescription medications without a prescription?
- Are pharmacists or others paid for advice on medicines at the point of supply? If so please describe including if there is any variation between practice settings (for example, if pharmacists in a primary care setting are reimbursed for counselling but community pharmacists are not).
- Roles of healthcare providers: Who has authority to prescribe? Are there different levels of provider status within professions (for example advanced practice nurse practitioners with prescribing authority)? Which healthcare providers are involved in reviews of medication profiles? In patient education?
- Policy: What policy is in place that supports the polypharmacy programme? If no polypharmacy programme is in place, briefly describe any other policies that influence medicines management.
- Legislation: What legislation is in place that directly or indirectly supports the polypharmacy programme? This might include items such as legislation defining who is a prescriber or mandating counselling on new prescription medicines. If there is no polypharmacy programme in place, describe how existing legislation would support or hinder implementation of a polypharmacy programme.

#### Role of government

*In this section, please address the role of national, territorial or regional government policies as they relate to the development or implementation of polypharmacy and adherence policies. If more than one level of government has authority or creates policies affecting the development and implementation of polypharmacy and adherence programmes include examples from each level.*

Please address the following points in your description:

- Are there official policies or programmes at the government level regarding polypharmacy and adherenceprogrammes that govern activities within your case study boundaries or support the polypharmacy programme?
  - Do these policies address polypharmacy, adherence, or both? How are each defined by the policy?
  - How do the policies on polypharmacy and adherence fit within the larger goals of the healthcare system?
- If no policies exist, describe where you looked to identify policies and what type of information you did find regarding medicines management in the elderly. For example, in Spain the Ministry of Health does not provide specific guidance on polypharmacy, but they do mention that polypharmacy management should be a part of comprehensive primary care services.
  - Provide a brief description of the types of medicines management documents that are available in your case study region regarding chronic disease management and the elderly.
- Why were the specific interventions introduced: What was the underlying rationale that set out the case for these interventions? Is there a clearly articulated vision for the programme? If there are no policies, what are the main priorities of the national health or local health system?
- How is policy information sent to the healthcare providers for action, and what if any monitoring of implementation is there? For example, in Scotland it is sent by the government to the health board leads and chief executives that requires them to take action on implementation and then report back.
- What incentives are in place (if any) for the implementation of these programmes? Are there any contractual requirements to provide polypharmacy reviews? Pay for performance?
- How is the impact of these policies measured? Are there systems or structures in place to monitor and evaluate the programme?

#### External organizations

*This section pertains to external organizations, such as health organisations andhealth boards responsible for healthcare provision, professional associations, scientific societies, licensing bodies, or other non-governmental organizations that may influence the development and implementation of polypharmacy and adherence programmes.*

- Are there other organisations outside of the government that play a role directly or indirectly establishing clinical protocols for polypharmacy and adherence within your programme?
- Why have these organisations become involved in polypharmacy and adherence?
- If so, please specify the organisations and the role they play. Specifically, provide the name, a description of their mission or activities and how they relate to polypharmacy and adherence programmes,their geographic scope (e.g. local or national) and how their activities interrelate with the institutions and government agencies that make up your case study.
- If no current policies exist, what role would outside organizations potentially play in developing guidelines and protocols related to polypharmacy and adherence in the elderly? Are there organizations working on related topics?

#### Health information and technology

##### Population Level Health Information

- What types of population level health indicators are available relative to polypharmacy and adherence? If a programme exists, how are these utilised (or not utilised) in your programme?
  - If so who has access to this data and why?
  - Specify if any of the systems referred to in the report are public or private.
- Is there any monitoring of prescribing patterns and the national, regional, or local level?
- Does the health system have the capacity to link patient specific data such as prescribed medications and comorbidities? What data specifically are available and how have they (or can they) be used to evaluate the impact of the intervention? At what level are the data available (city, county, regional, national)?
- Is there national or regional monitoring of prescription medications already undertaken, and for what purpose (for example, monitoring the cost and volume of medicines or for research)?

##### Patient Records

- Are patient records available electronically throughout the healthcare system?
- Do individuals in different institutions have access to the same information?
- Does each health care provider involved in polypharmacy management have access to the clinical patient records?
- Do any health care providers have limited access to patient information (for example some community pharmacists might not have access to laboratory values)?
- Do healthcare providers in the outpatient and inpatient have access to the same information? Are electronic patient records integrated throughout different healthcare settings?
- How are electronic patient records utilised in the polypharmacy and adherence programme?
- Do patients have access to their data? Are there any tools (such as aps for smartphones) to help them access their data?

##### Electronic Prescribing

- Does the health system utilize electronic prescribing?
- Is there an electronic database of dispensed medications?
- Who has access to prescribing and dispensing records?
- How is electronic prescribing utilised in the polypharmacy and adherence programme?

##### Integration and Future Plans

- Describe in general how information flows between different electronic health information systems.
- What, if any, new electronic health information technologies will your health care system be adopting within the next 2-3 years and how will this affect the polypharmacy and adherence programme?

### Clinical Decision Aids

- Do clinicians undertaking the medication reviews have access to clinical decision aids to aid in the selection of appropriate drug therapy?
  - If so, please describe the type of aid and how it is accessed (e.g. via smart phone, computer, etc.).
- What if any impact does this have on policy?
- Are any support tools available for patients?

### Institutional level

*The goal of the following section is to obtain a detailed description of the polypharmacy and adherence programme within your case study at the institutional level. This should be completed for each institution included in the case study.*

| ★ | *Partners with small pilot programmes may have difficulty addressing all of these points in this section. Regardless of the type of programme you have, please attempt to address each item below and make a note of any topics that you were unable to find.* |
| --- | --- |

### Why

- Why were the specific interventions introduced: what was the underlying rationale that set out the case for these interventions?

#### Where

- Where does the intervention take place? Please include a brief description of the each institution including:
  - Type of institution (primary care, tertiary teaching hospital, etc.)
  - Ownership and management (public, private, or mixture of both)
  - Urban or rural setting
  - Numbers of patients served*
  - Number of health care providers practicing at the institution*

* *If available, these data should also include the proportion of those patients and health care providers participating in or eligible for the polypharmacy programme. For example, you may have a 600 bed hospital but only patients on the 30 bed geriatric unit are targeted for the polypharmacy programme. Including both numbers will provide a better understanding of the resources devoted to the polypharmacy programme.*

#### What

- Is there a definition of polypharmacy and adherence at your institution?
- At the various institutions within your case study, are there policies or practice manuals in place regarding polypharmacy and adherence? If your case study includes more than one institution, such as a hospital and long-term care facility, address if there is a policy for each individual institution. If you have adopted guidelines, or based the development of your guidelines, on those from an outside institution such as a scientific society, please include that information here.
- Specifics of the guidelines:
- Does the guideline outline a clear drug review process? If it does what is it?

Does the guideline contain tools or advice to assist drug review?

If there are tools what are they (e.g. STOP STARTT)?

Does the guideline specify who should receive a polypharmacy review?

Does the guideline contain any specific information on high risk medications to target?

Does the guide contain any information on drug efficacy?

Are there elements of the guideline that specifically seek to lead to a patient centred / patient specific review (if so what are they)?

Does the guideline make use of worked examples?

- Were any training materials provided to staff prior to or during the development and implementation of the programme?If there were training materials what methods were used? Example might include written material, workshops, in-services, or online courses. Are any of these training activities ongoing?
- Is any information on the programme provided to patients? This might include items such as in-person counselling, education on specific medications or written information on their medication plan.
- Is any information, training, or support provided to carers?

#### Who

- Who provides the intervention? Please specify if it is a multidisciplinary team(specify team members e.g. physicians, pharmacists, nurses, or other providers), an individual practitioner, or a mix of the two. If the intervention occurs on more than one occasion (for example during a hospital admission and then in the primary care setting) specify who provides the intervention in each setting.
- What is their expertise related to this polypharmacy?

#### When and how much

- When does the intervention occur?
  - In person, such as during hospital rounds, at discharge, or during a regularly scheduled primary care visit.
  - On the phone
  - Virtual setting
  - Other
- How often does the intervention occur (e.g. once during hospital admission, on an ongoing basis during primary care visits). Please describe both the frequency and the location of medication reviews.
- Approximately how much health care provider time is spent on each intervention?

#### How

- How are patients selected to receive the intervention? Criteria may include items such as age, number of prescription medications, number of comorbid conditionsor the complexity of patient (explain how complex patients are identified and defined), absence or presence of frailty (as defined by your institution or practice setting), high-risk medications, patients with potentially inappropriate prescribing,or a combination of the above.
- Are patients at high risk of adverse events from the medicines prioritised for review?
- What specific services are provided as part of the intervention? Please provide a brief description.Examples of services include a medication profile review by a pharmacist or other health care provider, patient education, or team education. Please provide a description of each service.
- If a medication review is conducted, what were the goals of the review?General therapeutic review? Medication reconciliation? De-prescribing? Assess adherence?
- How is the information communicated to team members? Examples of communication methods include at the point of decision making (for example as part of rounds), as part of multidisciplinary case conference or post decision making such as with a fax or text message, written notes in medical chart with suggested changed or simply as an oral consult with the prescriber.
- How is information communicated between different levels of care, such as between a nursing home and hospital? Or between primary and secondarycare or between health and social care?
- How is information communicated to patients? Examples include verbal counselling, written prescriptions or written medication plans.
- Are pharmacists a part of the programme? If so, in what way? Please describe the practice settings of pharmacists involved in the programme (e.g. hospital pharmacists, community pharmacists, or pharmacists in general practice offices)
- If pharmacists are involved, is any training or certification required for participation?

### Tailoring

- Is the intervention designed to be individualised to specific patient needs? If so, in what ways was it individualised?
- How are the patient’s goals and therapy objectives incorporated into the review and subsequent care plan? How is this documented?

Outcome measures

- Has the intervention been measured?
  - If so, how? Examples might include efficacy outcomes such as the medication appropriateness index, markers of prescribing appropriateness, adverse drug events avoided, patient satisfaction, safety indicators such as hospitalizations avoided, or the efficiency or economic impact of the programme. Include all types of outcomes that have been evaluated.
  - What system or structures exists for capturing intervention effects?
- How much time was involved in the evaluation process? Were additional staff required for the evaluation phase?
- Has there been any evaluation of the programme published within the last five years in peer-reviewed publications? Please include a PDF of the document.

## Interview Guide

***Introduction***

Hello, my name is XX and I am from XXX. Thank you for agreeing to be interviewed as a part of the SIMPATHY project—we appreciate you contributing your time to our work.

As was explained in the introductory email, SIMPATHY is a consortium of 8 countries in the European Union with the goal of promoting innovation around polypharmacy and adherence programmes in older people. As part of this project, we are conducting case studies in different countries to help us better understand what polypharmacy programmes do or do not exist, but also what facilitated or hindered the development, implementation, and sustainability of these programmes. The goal of this interview is to learn more about the state of polypharmacy management and adherence in XX location.

This interview should not last longer than an hour. With your permission, we’d like to record the interview. All of the recordings and the notes I take will be used exclusively in this study, and will remain anonymous and confidential.

Before we start, may I ask you to sign this consent form that outlines the information that I’ve just explained? Please take your time to read it before you sign.

**1) Do you have any questions before we begin?**

***2)To begin with, can you give me a brief description of your role within [name of institution] and how and why you are involved in the polypharmacy programme [or medicines management policies]?***

- I want to be sure that we’re all talking about the same thing, so I´m going to define a few terms. When I say ***medicines management***, I’m referring to the entire process of how medicines are selected, procured, delivered, prescribed, administered and reviewed to optimise patient outcomes. This not only includes clinical activities, but also the development of guidelines and policies to govern the process. ***Inappropriate polypharmacy*** is when a patient is on multiple medications (usually five or more), and the risk of harm from those medicines outweighs the benefits. Sometimes polypharmacy is appropriate and indicated, like when a patient has multiple chronic conditions, but many times it is not appropriate and can result in patient harm. A polypharmacy management programme is a systematic medicines management programme focused on optimizing the drug therapy of patients on multiple medications.
- Are you aware about the issue of inappropriate polypharmacy associated with multimorbidity? Do you think it affects you? Why (or why not) and how?

***Now I’d like to ask some questions about the decision making process.***

2a) In general, how would you say decisions are made in your work place? In your healthcare system?

- Are decisions collaborative, bottom-up, top down, structured, non-structured?
- Can you provide an example of how a decision in your practice setting is made, such as a decision about drug therapy [*modify this example as needed based on the expertise of your interview subject*]? Who participates in making this decision? What sources of information do they use? Is the decision by consensus or majority? How is the decision communicated to others?

2b) Can you describe the key characteristics of decision-making?

| ★ | *Interview instructions—select one of the follow scenarios below based on the maturity of your program. Option 1.1 is for existing programmes and option 1.2 is for non-existing programmes.* |
| --- | --- |

- 1. ***Now I’d like to talk to you about your polypharmacy management programme. Can you give me an overview of the programme, and why and how it got to where it is now?***
- How is the problem of polypharmacy articulated by your organisation or government, and
- Why does the government or organisation see the need to address it now?
- Would you say that there was a clearly articulated vision?
  1. ***Now I’d like to talk to you about medicines management in your country or institution. Can you give me an overview of how drug therapy is managed?***
- Has the problem of polypharmacy been articulated by your institution? If not, why do you think this is?
- Has there been any attempt to describe the benefits of polypharmacy and adherence management? An example of this might be an economic evaluation of the impact of non-adherence or inappropriate polypharmacy.
- How does a polypharmacy management or adherence plan fit within the goals of your institution, or, how does it not fit?
- Have there been any attempts to draft any proposals around polypharmacy management and adherence? How have these been received?

***One of our goals in this project is to understand how different polypharmacy programmes were conceived, developed, and implemented, and if no programme exists, why this is. Now I’d like to get into some more specifics of your situation.***

| ★ | *Interview instructions—select one of the follow scenarios below based on the maturity of your program. Option 2.1 is for existing programmes and option 2.2 is for non-existing programmes.* |
| --- | --- |

- 1. ***I’d like to ask some questions about the initial planning phase.***
- Can you please describe the key characteristics of the planning phase?
- How is planning addressed in your organisation and who is responsible?
- Looking back to the start of the programme, how were the benefits assessed prior to implementation?
- What economic evaluation, if any, was used in the planning and why?
- Was a business case made for the adoption of the programme? If so please describe why.
  1. **I’d like you to think about implementing a new polypharmacy programme.**
- Can you please describe some of the key steps that would be necessary for the initial planning phase? For example, the need to develop standardized practice guidelines, or create a working group on the issue.
- In your organization, who would be responsible for this type of initiative?
- What elements of feasibility (bottlenecks/ enablers) would be used to determine if this type of programme would go forward?
- Would an economic evaluation typically be part of the planning process?
- Does a polypharmacy management programme make sense in the context of the ongoing work at your institution? Does it fit with your goals and objectives? Why or why not?

***[CONTEXT FOR INTERVIEWER—USE AS PROMPT IF NEEDED IN EITHER SCENARIO]***

***For example, in Scotland, initially individual business cases would have been prepared that would explain the benefits to individual regional board. When the first national guidance was produced evidence from the boards with economic benefits was gathered to help the boards build the case for undertaking the work. For further information see Scottish polypharmacy guidance version 1.***

| ★ | *Interview instructions—the following section refers to the implementation and integration of a programme into an organization. Two sets of questions are provided under each heading, one for existing programmes and one for potential future programmes. Only use one set during the interview depending on the status of your programme* |
| --- | --- |

***Now I’d like to learn some more about how the programme was introduced into the organization [OR how a future programme would be introduced to your organization].***

***Existing Programmes***

- How was the programme described to the clinicians who would be responsible for implementing it?
- How were the benefits described?
- How did it relate to your organizational goals?
- Was there resistance to change? From individuals or from larger groups, such as from a specific group of healthcare providers or policymakers.

***Future Programmes***

- Who would be responsible for describing the programme to clinicians responsible for implementing it?
- Which target population would you address for the implementation of the program? Perhaps a chronic condition like diabetes or an age group such as frail elderly?
- Do you anticipate that there would be resistance? If so, from which groups?
- Who are the major stakeholders, or, who are the people whose lives would be most affected by the implementation of a polypharmacy management programme?
  - Would these people be facilitators or detractors from the programme?
  - How would you work with the detractors?
  - How would the programme benefit from facilitators?

***The next group of questions involve the implementation of the programme.***

***Existing Programmes***

- How would you say that the programme was initially received by those implementing it?
  - Was there buy in or were people sceptical of the benefits?
- Were there any key individuals or champions involved in the implementation?
- What type of coalitions, management groups, or teams were formed to help implement the programme? Who would you say had the primary leadership role?
- Have the polypharmacy reviews enabled more multidisciplinary interactions with the pharmacists?
- Are the patients supported to make decisions from the review?
- Has support for the intervention changed over time?

***Future Programmes***

- How do you think this type of programme would be received by those responsible for implementing it?
- Who would be the champions necessary for its success?
- How would you set up a pilot to test and implement the model?
- What type of coalitions, management groups or teams do you think would help with the implementation? Who do you think should coordinate such an initiative?
- What role do you see for patients in the implementation?

***Now I want to learn a little more about how you integrated the programme into the existing work load of your clinicians [OR how you would integrate a programme into the existing work load of your clinicians].***

***Existing Programmes***

- What type of training was provided?
- If training was provided who was this for and why?
- How did the programme fit with existing tasks?
- Were new staffing patterns required? Restructure departments? Modify workflow?
- Were additional resources required?
- Was there an initial trial run or pilot programme prior to full-scale implementation?
- How many units were involved in the initial implementation? One unit geriatric ward in a hospital? The whole institution? More than one institution? Has it been expanded to include more units?
- What elements of the healthcare system were barriers to change?
- What elements helped?

***Future Programmes***

- What type of training do you think would be necessary for the aforementioned target populations this type of initiative to succeed? If so, for whom?
- How do you see this new programme fitting in with existing tasks, such as training/literacy/programs/activities?
- Do you think that a new staffing pattern would be required? What other changes to personnel management do you think would be required?
- Would this require additional resources?
- What do you think would be the most successful strategy for implementing a new programme regarding the location and size of the programme? For example, would you suggest a trial run in a hospital ward first? In the context of a stepwise approach?
- What elements of your healthcare system do you think would be potential barriers or facilitators to implementing this type of programme?

***Finally I want to talk to you about the evaluation of your programme [OR of a future programme].***

***Existing Programmes***

***Can you summarise the results of your programme?***

- What type of short term monitoring of outcomes was done? How were these results shared with staff?
- Why was monitoring undertaken?
- How are the effects of the programme evaluated?
- Were there any unintended outcomes, either positive or negative? These might include things like additional paperwork, improved relationships between providers, or negative clinical outcomes from aggressive deprescribing.
- Is staff motivation an issue that needs support? Why or why not? How has staff motivation been sustained?

***Future Programmes***

- How would you define and monitor the short term outcomes of such a programme? Are there mechanisms in place to share this type of information with your staff?
- How else would you define and evaluate the success of this type of programme?
- Do you think that staff motivation over the long term will be an issue that needs support?

***I’d like to understand a little more about the practice environment, especially around who makes drug therapy decisions.***

- Which health care providers have prescribing authority in your programme?
- How healthcare decisions are typically made? For example, by a multidisciplinary team or individual practitioners?
- How are prescribing decisions made and why?
- Would you describe the environment within your programme as collaborative? Hierarchical? Or in other terms (please explain)? How has that shaped this programme?
- Outside of physicians, do other health care providers such as nurses or pharmacists have increased clinical involvement in patient care such as prescribing authority, responsibility for patient education, or the development of drug therapy plans?

***Now I want to talk a little about your plans for the future.***

- Looking forward over the next 2-3 years, what are the goals and objectives for the programme [OR for your institution regarding medicines management]? Please describe why these have been chosen.
- What will be required to achieve these goals?

| ★ | *Interview instructions—the following group of questions only applies to countries with existing programmes.* |
| --- | --- |

***Finally, if you were advising someone on the development of a polypharmacy programme, what key piece of advice would you give them? Is there anything you would have done differently in your programme, or anything that you see as essential to the success of a polypharmacy management programme?***

***Thank you so much for your participation. Before we end, is there anything that I did not ask you about that you would like to explain about the development and implementation of your polypharmacy programme [OR about the potential development of a polypharmacy and adherence programme]?***

***We may want to consult with you in the process of writing up the case studies to seek clarification on specific points – we hope you will be amenable to this?***

## Focus group discussion guide

Hello, my name is [Moderator’s name] and this is my colleague [Note taker’s name].  Welcome to the discussion.  Today I would like to discuss your opinions of the SIMPATHY report regarding the management of polypharmacy and adherence. Everything you say is important to us and will help us determine if our findings reflect the true situation that patients and health care providers experience.  Please feel free to speak openly and use any language or words. There are no right or wrong answers.  Your name will not be written anywhere, which means that no one will know it was you who said something. You can choose to stop participating in this discussion at any time and you can choose not to respond to any question you don’t want to answer, but we hope you will feel free to contribute.

Since this discussion is very important to us, we would like to audio record it, with your permission (confirm their consent). My colleague [Note taker] will also be taking notes to make sure that we do not miss any important things that we will discuss today.   The recording and notes will be kept private and safe.  The discussion will take about 90 minutes.  Do you have any questions at this point?   We are now turning on the audio recorder.

**Brief Introduction and Context Setting:**

I’d like to briefly summarize some of the main points from the report. *THIS WILL NEED TO BE DEVELOPED BY EACH PARTNER BASED ON THE RESULTS OF THEIR PARTICULAR REPORT.*

**Initial Reaction:**

1. *Ask each participant to write down three words or phrases that describe their initial reaction to the repot contents. [Alternatively, or additionally at some point, ask each participant to write down the two strongest points of the report and the two points that need improvement or clarification].  Have participants share what they wrote down.*

**Individual Experience:**

1. How would you say the description of polypharmacy management compares to your experience?
   1. What about the report fits with your experience?
   2. Are there aspects of the report that don’t fit with your experience?

*Questions 3-4 are for patients or care givers only*

1. Were you aware that there is an initiative in XX focusing on polypharmacy, or people taking many medications
2. Has a doctor or other health care provider such as a nurse or pharmacist ever talked to you about your medicines plan?
   1. If so, what did they talk to you about?
   2. How did your experience compare to the one described in the report?

*Question five applies to policy makers and managers*

Now I’d like to ask your opinion about some of the specifics in the report.

1. How does the description of the development and implementation of the [NAME OF PROGRAMME] polypharmacy programme match with your experience?
   1. The description of the evaluation?
   2. Management techniques described?

**General Feedback:**

1. Is there anything in the report that you feel is not accurate?
2. What is the strongest aspect of the report?
3. Is there anything that you feel we have missed or that should be added to the report?

**Summarize key points prior to closing session:**

Before we finish I’d like to summarize what I heard as your main points regarding this report.

**Conclusion:**

Thank you for your time. If you have any additional questions or comments, you may contact [NAME], Study Coordinator, at PHONE or EMAIL.
